# The effect of cyberchondria on anxiety, depression and quality of life during COVID-19: the mediational role of obsessive-compulsive symptoms and Internet addiction

**DOI:** 10.1016/j.heliyon.2022.e09437

**Published:** 2022-05-14

**Authors:** Federica Ambrosini, Roberto Truzoli, Matteo Vismara, Daniele Vitella, Roberta Biolcati

**Affiliations:** a Department of Education Studies “Giovanni Maria Bertin”, University of Bologna, Italy; b Department of Biomedical Sciences and Clinics Luigi Sacco, Faculty of Medicine and Surgery, University of Milan, Italy; c Aldo Ravelli Center for Neurotechnology and Brain Therapeutics, University of Milan, Italy; d University of Milan, Italy

**Keywords:** Cyberchondria, Internet addiction, Compulsivity, Anxiety, Depression, Quality of life

## Abstract

Since the global pandemic of the coronavirus disease 2019 (COVID-19), online health information-seeking behaviors have notably increased. Cyberchondria can be a vulnerability factor for the worsening of anxiety-depressive symptoms and quality of life. The current study aims to understand the predictive effect of cyberchondria on health anxiety, anxiety, depression and quality of life considering the mediating effect of obsessive-compulsive symptoms and Internet addiction and the moderating effect of COVID anxiety. 572 Italian participants (66% female; Mean age = 34; SD = 15) took part in a cross-sectional online survey involving CSS-12, MOCQ-R, IAT, SHAI, HADS, WHOQoL-BREF and CAS. Mediation and moderation analyses were conducted. Obsessive-compulsive symptoms and Internet addiction were found to partially mediate the cyberchondria-health anxiety and the cyberchondria-anxiety links and to totally mediate the cyberchondria-depression and the cyberchondria-quality of life links. COVID anxiety was found to moderate the relationship between cyberchondria and anxiety. The findings suggest that compulsivity may have a key role in the explanation of the underlying mechanisms of cyberchondria. Healthcare practitioners should provide additional support for individuals with cyberchondria. As such, cyberchondria is a contributing factor to the exacerbation of anxiety-depressive disorders and may impact on the quality of life.

## 1 Introduction

Since the global pandemic of the coronavirus disease 2019 (COVID-19), online health information-seeking behaviors have increased (Bento et al., 2020) concurrently with the growing need to achieve fast and updated details about the etiology, presentation and prevention of the disease (Renu et al., 2021). Albeit with some differences across countries (Wang et al., 2020, 2021a, 2021b), the increase of incoming information has someway facilitated the understanding of the disease's seriousness (Jokic-Begic et al., 2020) and the engagement of preventive behaviors against it (Liu, 2020). However, the overload of information from all types of media has also intensified several psychological consequences, common to pandemics and epidemics (Bish and Michie, 2010; Lau et al., 2010; Sauer et al., 2020), such as an increase in health anxiety, medical concerns and distress (Garfin et al., 2020).

In this scenario, cyberchondria could have a detrimental effect on mental health and quality of life.

A recent review provided a comprehensive working definition of cyberchondria, identified as “a pattern of excessive searching on the Internet for medical or health-related information with the following features: searching is compulsive, hard to resist and serves the purpose of seeking reassurance; initial relief, if obtained, through online searching is short-lived and anxiety or distress usually worsens during these searches and persists afterwards; online searching takes precedence over other interests or daily activities and continues or escalates despite the occurrence of negative consequences associated with the searching” (Vismara et al., 2020, p. 7).

As highlighted by Starcevic et al. (2020), this definition includes a “compulsive” component and emphasizes the similarities with behavioral addictions. Indeed, several studies highlighted the presence of obsessive-compulsive components in cyberchondria (Fergus, 2014; McElroy and Shevlin, 2014). According to the cognitive-behavioral model, preoccupations about health can elicit an information seeking behavior aimed at self-reassurance, which may be exhibited through compulsive searches on the Internet (Erdoğan and Hocaoğlu, 2020). However, the terminology and the content distribution of web search engines may lead the reader to focus on more serious and relatively rare conditions, with the potential to escalate health preoccupations and anxiety (White and Horvitz, 2009). This can elicit further excessive or dysfunctional health-related online searches, creating a vicious cycle difficult to interrupt (Jokic-Begic et al., 2020).

Compulsivity, characterized by the development of repetitive behaviors, often carried out to avoid adverse or anxiety-inducing outcomes, regardless of their consequences, at the cost of healthy rewarding actions, represents a key feature common to both obsessive-compulsive disorder and behavioral addictions (Figee et al., 2016). In cyberchondria, the urge to search despite the negative effects (i.e., time-spending, higher distress, neglect of commitments, interpersonal conflict, problems with health professionals, and increasing use of health care services) (Khazaal et al., 2021) and the consequent loss of control over Internet use, and in particular health information seeking, may be features associated with a form of behavioral addiction (Billieux et al., 2016; Kardefelt-Winther et al., 2017). Previous studies (Doherty-Torstrick et al., 2016; Ivanova, 2013) have already considered cyberchondria as a specifically compulsive form of problematic Internet use characterized by health concerns, suggesting potential links between cyberchondria and Internet addiction, which seem to be confirmed in a recent study by Mrayyan et al. (2022). Other scholars showed that individuals characterized by a problematic use of the Internet, which causes preoccupations, significant distress or functional impairment (Shapira et al., 2003), tend to show a more severe cyberchondria, regardless of age, gender, current reported medical status, negative affect or health anxiety (Fergus and Spada, 2017).

Therefore, even though most of the studies on this emerging condition have concentrated on the overlapping features between cyberchondria and health anxiety (e.g., Erdoğan and Hocaoğlu, 2020), the strength of this relationship is still a matter of debate (Mathes et al., 2018) and lines of research suggest that they may be considered as distinct constructs (Menon et al., 2020; Schenkel et al., 2021). The latter findings implied that the evaluation of cyberchondria must be fulfilled also in the absence of pre-disposing health anxiety and it may be even considered as a vulnerability factor for health anxiety (Menon et al., 2020; Singh and Brown, 2016). Indeed, excessive online health information seeking could worsen the symptom expression of more severe mental illnesses such as anxiety and depressive disorders, worsening the quality of life (Mathes et al., 2018).

Regarding the impact of online health information seeking on anxiety, depression and quality of life, research is still in progress. Using the Internet for health purposes resulted associated with slight increases in depression in a previous longitudinal study (Bessière et al., 2010), whereas Starcevic et al. (2019) showed a weak relationship between cyberchondria and depression, and cyberchondria and general anxiety. Anyway, a recent study by Arsenakis et al. (2021), found that also depressive symptoms seem to be associated with cyberchondria. Only few previous studies highlighted that cyberchondria may also affect psychosocial functioning and quality of life (Mathes et al., 2018; Rahme et al., 2021). However, further research is needed.

With the purpose of unveiling the relationship between cyberchondria and a set of constructs, some studies have recently focused on the building of mediation and moderation models (Ivanova and Karabeliova, 2014; Bajcar and Babiak, 2019; Batıgün et al., 2020; Abdelsattar et al., 2021; Han et al., 2021; Rahme et al., 2021), mainly considering cyberchondria as a mediator or as a dependent variable instead of exploring its impact on specific outcomes.

In absence of a consensus-based definition and conceptualization of cyberchondria, deepening its effects on better-known psychological symptoms and quality of life may be useful to underline clinical and public health implications of cyberchondria during the pandemic (Starcevic et al., 2020). Thus, this study expands the paucity of literature examining the extent to which and through what cyberchondria may be a public health concern, with regard to its impact on psychological suffering and quality of life. Specifically, the present study focused on the impact of cyberchondria on several mental health outcomes (i.e., health anxiety, anxiety, depression) and quality of life, exploring the mediational roles of compulsive features such as obsessive-compulsive symptoms and Internet addiction. Moreover, considering that previous evidence showed that COVID anxiety can have an impact on cyberchondria (Wu et al., 2021), COVID anxiety was analyzed as a potential moderator of the relationship between cyberchondria and health anxiety, anxiety, depression and quality of life.

Hence, the aims of this study are 1) to examine levels of health anxiety, anxiety, depression, quality of life, COVID anxiety and cyberchondria in a set of data collected during the third wave of COVID-19; 2) to test the model with cyberchondria as predictor of a worsening in health anxiety, anxiety, depression and quality of life, with the mediation of obsessive-compulsive symptoms and Internet addiction; 3) to analyze if COVID anxiety moderates the relationship between cyberchondria and health anxiety, anxiety, depression and quality of life.

## 2 Material and methods

### 2.1 Participants and procedure

This cross-sectional study is part of a wider research carried out by the authors investigating health-related information search and Cyberchondria in the Italian general population during the COVID-19 pandemic (Vismara et al., 2021). Data were collected from February 1^st^, 2021 to April 30^th^ 2021, during the third wave of COVID-19 pandemic, throughout which specific restrictive measures were implemented by the Italian Government (including the social distancing, the curfew at 22 p.m., the ban to entry/exit from/to different regions until April 26^th^ except for extraordinary and documented reasons). Hence, the study was conducted using a snowball sampling technique. A link to the online survey on the Google Form web domain was sent via e-mail, social networks and instant messaging applications to adult members of the Italian general population (the inclusion criteria to participate were age ≥18 and currently living in Italy). Each respondent could answer the survey only once.

The present study was approved by the Ethical Committee of the University of Milan (Approval number: 27/21) and was performed in accordance with the ethical standards as laid down in the 1964 Declaration of Helsinki and its later amendments. Participants gave their informed consent by clicking the Start button on the online survey and entering the next page.

### 2.2 Measures

The *Cyberchondria Severity Scale short form* (CSS-12, McElroy et al., 2019; Soraci et al., 2020) was used to assess the severity of anxiety and distress associated with health-related online searches. This 12-item self-report scale rates each item on a 5-point scale ranging from 1 (never) to 5 (always).

The Schedule 9 of CBA 2.0 (Sanavio et al., 1986), the *reduced form of the Maudsley Obsessional-Compulsive Questionnaire* (MOCQ-R), was used to evaluate the severity of obsessive-compulsive symptoms in three domains: checking, cleaning and doubting-ruminating. The schedule is composed of 21 dichotomous items (true/false).

The *Internet Addiction Test* (IAT, Ferraro et al., 2006; Young, 1999) was used to evaluate the severity of individual and social impairment in quality of life because of a compensatory usage of the Internet. The test is composed of 20 items, rated on a 5-point Likert scale from 1 (never) to 5 (always).

The *Short Health Anxiety Inventory* (SHAI, Salkovskis et al., 2002) was used to assess health anxiety, regardless of physical health status. It is composed of 18 items, rated from 0 to 3, evaluating worries about health, awareness of bodily sensations or changes and the concerns about consequences of having illnesses.

The *Hospital Anxiety and Depression Scale* (HADS, Costantini et al., 1999; Zigmond and Snaith, 1983) is a 14-item self-report scale rated on a 4-point Likert scale (0–3), comprehensive of two subscales (each composed of 7 items) evaluating the severity of emotional disorders related to anxiety and depression. Thus, the present study used the two scores at the subscales as measures of the severity of anxious and depressive symptoms.

The *World Health Organization Quality of Life—BREF* (WHOQoL, De Girolamo et al., 2000; World Health Organization, 1998) was used to assess the quality of life. It includes 26 items and is rated on a 5-point Likert scale (1–4).

The *Coronavirus Anxiety Scale* (CAS, Lee, 2020) is a short 5-item screener for dysfunctional anxiety related to COVID-19, scored on a 5-point Likert scale (from 0 = “Not at all” to 4 = “Nearly every day over the last two weeks”).

All the variables included in the present study showed good internal consistencies (Cronbach α ranges from .77 to .91).

### 2.3 Statistical analysis

Data analysis was performed with SPSS 26 (IBM Corp., 2019). There were no missing values in the analyzed variables. Based on the variables' distribution, t-test and Mann-Whitney test were used to examine the effect of sex, occupational status and presence of physical and psychiatric comorbidities on the psychological variables. Linear regression and generalized linear model were used to examine the impact of age on the psychological variables. Pearson and Spearman zero-order correlations were used to investigate the relationships between cyberchondria and the study variables. Mediation and moderation effects were examined using PROCESS macro for SPSS v.3.4 (Hayes, 2018), an observed-variable modeling tool used for estimating direct and indirect effects in single and multiple mediator models, interactions in moderation models and conditional indirect effects in moderated mediation models. Unlike other methods able to do path analysis with observed variables such as structural equation modeling, PROCESS estimates the parameters using ordinary least squares (OLS) regression and perform each equation independently, so that the estimation of the regression parameters in one of the equations has not influence on the parameters’ estimation of other equations of the model (Hayes et al., 2017). Moreover, PROCESS does not offer omnibus measures of model fit (Hayes et al., 2017).

OLS assumptions must be met to conduct mediation and moderation analysis with PROCESS (Hayes, 2018). Therefore, eight hierarchical regression analyses were conducted (one for each mediation and moderation model tested). Independence of residuals, residuals’ normal distribution, absence of multicollinearity, and homoscedasticity were evaluated using the Durbin-Watson statistics, histograms and p-p plot of residuals, the Variance Inflation Factor (VIF) and scatterplots of standardized residuals vs predicted values, respectively. The results of preliminary regression analysis are shown in Supplementary contents.

Model 4 of PROCESS was used to test the indirect effect of cyberchondria on health anxiety, anxiety, depression and quality of life through obsessive-compulsive symptoms and Internet addiction. Four mediation analyses were performed, one for each dependent variable (health anxiety, anxiety, depression, quality of life). COVID anxiety, sex, age, occupation and the presence of physical/psychiatric comorbidities were considered as covariates, to check for any confounding effects.

Lastly, the moderating effect of COVID anxiety on the relationship between cyberchondria and the dependent variables and its combined mediating effects were examined in four integrated models performed with PROCESS Model 5. Sex, age, occupational status and the presence of physical/psychiatric comorbidities were set as covariate, to check for any confounding effects. Scores at CSS-12 and at CAS were mean centered prior to analysis. The conventional 'pick-a-point' approach was used to test the moderation effect, choosing the 16^th^, 50^th^ and 84^th^ percentiles as representative values of the moderator and then estimating the effect of the focal predictor at those values, as suggested by Hayes (2018). Interactions were probed with α < .05.

In the Model 4 and Model 5 the percentile bootstrap confidence intervals (CIs) with 10,000 replications and a 95% confidence interval were used to investigate the indirect effects. A common seed for the bootstrapping was set. Statistical significance was established when zero was not included in the lower and upper levels of the CIs (Hayes, 2018).

## 3 Results

### 3.1 Sample characteristics

The sample was composed of 572 Italian adults living in Italy, 375 females (66%), aged from 18 to 77 (mean age = 34 years old, SD = 15). No significant differences between males and females based on age were found (F_1,570_ = .024; p = .877) (see Table 1).

**Table 1:** Demographic characteristics of the sample.

	Respondents *n* = 572
**Age***mean (SD; range)*	33.6 (14.6; 18–77)
**Gender***n M/F (%M)*	197/375 (34.4)
**Occupational status***n (%)*	
Employed	241 (42.1)
Retired	35 (6.1)
Student	264 (46.2)
Housewife	13 (2.3)
Not employed	19 (3.3)
**Comorbid disease***n (%)*	
No	419 (73.3)
Yes	153 (26.7)
**Psychiatric comorbid disorder***n (%)*	
No	530 (92.7)
Yes	42 (7.3)

### 3.2 Differences in psychological variables based on gender, age, occupational status and physical/psychiatric comorbidities and zero-order correlations

Compared to males, females exhibited higher levels of cyberchondria, anxiety, depression and COVID anxiety (see Table 2). Instead, greater levels of internet addiction were found in males compared to females. Younger participants showed higher levels of cyberchondria, obsessive-compulsive symptoms, internet addiction, health anxiety, anxiety and depression. Students compared to Not students showed higher levels of cyberchondria, internet addiction, anxiety and depression. Participants with physical comorbidities tended to show higher levels of cyberchondria, obsessive-compulsive symptoms, health anxiety, anxiety, depression and worse quality of life. Participants who declared to suffer from a psychiatric disorder showed worse levels of cyberchondria, obsessive-compulsive symptoms, internet addiction, health anxiety, anxiety, depression and quality of life

**Table 2:** Differences based on age, sex, occupation and presence of physical and psychiatric comorbidities.

	Sex			Age		Occupation			Physical comorbidities			Psychiatric comorbidities		
	Males	Females	*U*	*b*	*Wald χ2*	Student	Not student	*U*	Yes	No	*U*	Yes	No	*U*
	*Mean rank*	*Mean rank*				*Mean rank*	*Mean rank*		*Mean rank*	*Mean rank*		*Mean rank*	*Mean rank*	
CSS-12	265.6	297.5	32813.5∗	-.003	9.30∗∗	301.9	273.3	36590.5∗	313.3	276.7	27955.5∗	376.0	279.4	7371.00∗∗∗
CAS	222.7	320.0	24368.5∗∗∗	-.009	9.185∗∗	297.5	277.1	37765.5	287.7	286.1	31867.5	325.6	283.4	9486.00
	**Sex**			**Age**		**Occupation**			**Physical comorbidities**			**Psychiatric comorbidities**		
	***Males***	***Females***	*t*	*b*	*t*	***Student***	***Not student***	*t*	***Yes***	***No***	*t*	***Yes***	***No***	*t*
	*M (SD)*	*M (SD)*				*M (SD)*	*M (SD)*		*M (SD)*	*M (SD)*		*M (SD)*	*M (SD)*	
MOCQ-R	5.5 (4.2)	5.1 (3.8)	1.19	-.034	-2.99∗∗	5.5 (4.1)	5.0 (3.9)	-1.51	6.6 (4.3)	4.8 (3.7)	-4.58∗∗∗	8.6 (4.8)	5.0 (3.8)	-4.87∗∗∗
IAT	39.5 (12.6)	36.3 (11.4)	2.92∗∗	-.283	-8.84∗∗∗	40.2 (11.6)	35.0 (11.7)	-5.33∗∗∗	39.0 (13.7)	36.8 (11.2)	-1.79	44.2 (15.0)	36.9 (11.5)	-3.09∗∗
SHAI	15.3 (8.4)	15.9 (7.7)	-.75	-.051	-2.24∗	16.1 (7.4)	15.3 (8.4)	-1.31	18.5 (9.1)	14.7 (7.2)	-4.66∗∗∗	22.7 (9.5)	15.1 (7.5)	-5.04∗∗∗
HADS-A	6.1 (4.3)	7.4 (4.1)	-3.57∗∗∗	-.068	-5.81∗∗∗	7.7 (4.3)	6.3 (3.9)	-4.07∗∗∗	8.1 (4.6)	6.6 (3.9)	-3.83∗∗∗	11.1 (4.4)	6.7 (4.0)	-6.83∗∗∗
HADS-D	3.9 (3.1)	4.5 (3.3)	-2.09∗	-.032	-3.43∗∗	4.8 (3.4)	3.9 (3.1)	-3.22∗∗	5.2 (3.6)	4.0 (3.1)	-3.74∗∗∗	6.7 (3.6)	4.1 (3.1)	-5.21∗∗∗
WHOQoL	88.0 (10.6)	89.8 (10.7)	-1.86	-.005	-.15	89.0 (11.2)	89.3 (10.3)	.35	86.0 (11.4)	90.3 (10.2)	4.27∗∗∗	79.9 (13.0)	89.9 (10.2)	4.85∗∗∗

*Note.* ∗*p* < .05; ∗∗*p* < .005; ∗∗∗*p* < .001. CSS-12, Cyberchondria Severity Scale short version; MOCQ-R, reduced form of the Maudsley Obsessional-Compulsive Questionnaire; IAT, Internet Addiction Test; SHAI, Short Health Anxiety Inventory; HADS-A, Hospital Anxiety and Depression Scale, Anxiety subscale; HADS-S, Hospital Anxiety and Depression Scale, Depression subscale; WHOQoL, WHO Quality of Life-BREF; CAS, Coronavirus Anxiety Scale.

Statistically significant zero-order correlations were found between all variables tested in the mediation and moderation models (see Table 3).

**Table 3:** Descriptive statistics, Cronbach α and zero-order correlations among all the models’ variables.

	M (SD)	Range	α	CSS-12^a^	MOCQ-R^b^	IAT^b^	SHAI^b^	HADS-A^b^	HADS-D^b^	WHOQoL^b^	CAS^a^
CSS-12^a^	21.4 (7.9)	12–56	.88	-	.34∗∗∗	.33∗∗∗	.56∗∗∗	.38∗∗∗	.29∗∗∗	-.16∗∗∗	.32∗∗∗
MOCQ-R^b^	5.2 (4.0)	0–20	.81		-	.35∗∗∗	.42∗∗∗	.50∗∗∗	.38∗∗∗	-.36∗∗∗	.22∗∗∗
IAT^b^	37.4 (11.9)	20–84	.91			-	.36∗∗∗	.35∗∗∗	.35∗∗∗	-.28∗∗∗	.18∗∗∗
SHAI^b^	15.7 (7.9)	1–47	.89				-	.53∗∗∗	.42∗∗∗	-.31∗∗∗	.35∗∗∗
HADS-A^b^	7.0 (4.2)	0–20	.85					-	.65∗∗∗	-.49∗∗∗	.46∗∗∗
HADS-D^b^	4.3 (3.2)	0–16	.77						-	-.59∗∗∗	.32∗∗∗
WHOQoL^b^	89.2 (10.7)	53–119	.88							-	-.17∗∗∗
CAS^a^	2.6 (3.1)	0–18	.84								-

*Note.*^a^ Spearman rank correlation coefficient; ^b^ Pearson *r* correlation coefficient. ∗p < .05; ∗∗p < .005; ∗∗∗p < .001.

α, Cronbach alpha; CSS-12, Cyberchondria Severity Scale short version; MOCQ-R, reduced form of the Maudsley Obsessional-Compulsive Questionnaire; IAT, Internet Addiction Test; SHAI, Short Health Anxiety Inventory; HADS-A, Hospital Anxiety and Depression Scale, Anxiety subscale; HADS-D, Hospital Anxiety and Depression Scale, Depression subscale; WHOQoL, WHO Quality of Life-BREF; CAS, Coronavirus Anxiety Scale.

### 3.3 Mediating effect of obsessive-compulsive symptoms and internet addiction

Results of Model 4 of Process indicated that, after controlling for gender, age, occupation, physical/psychiatric comorbidities and COVID anxiety, cyberchondria positively influenced obsessive-compulsive symptoms and Internet addiction.

**Figure 1:**
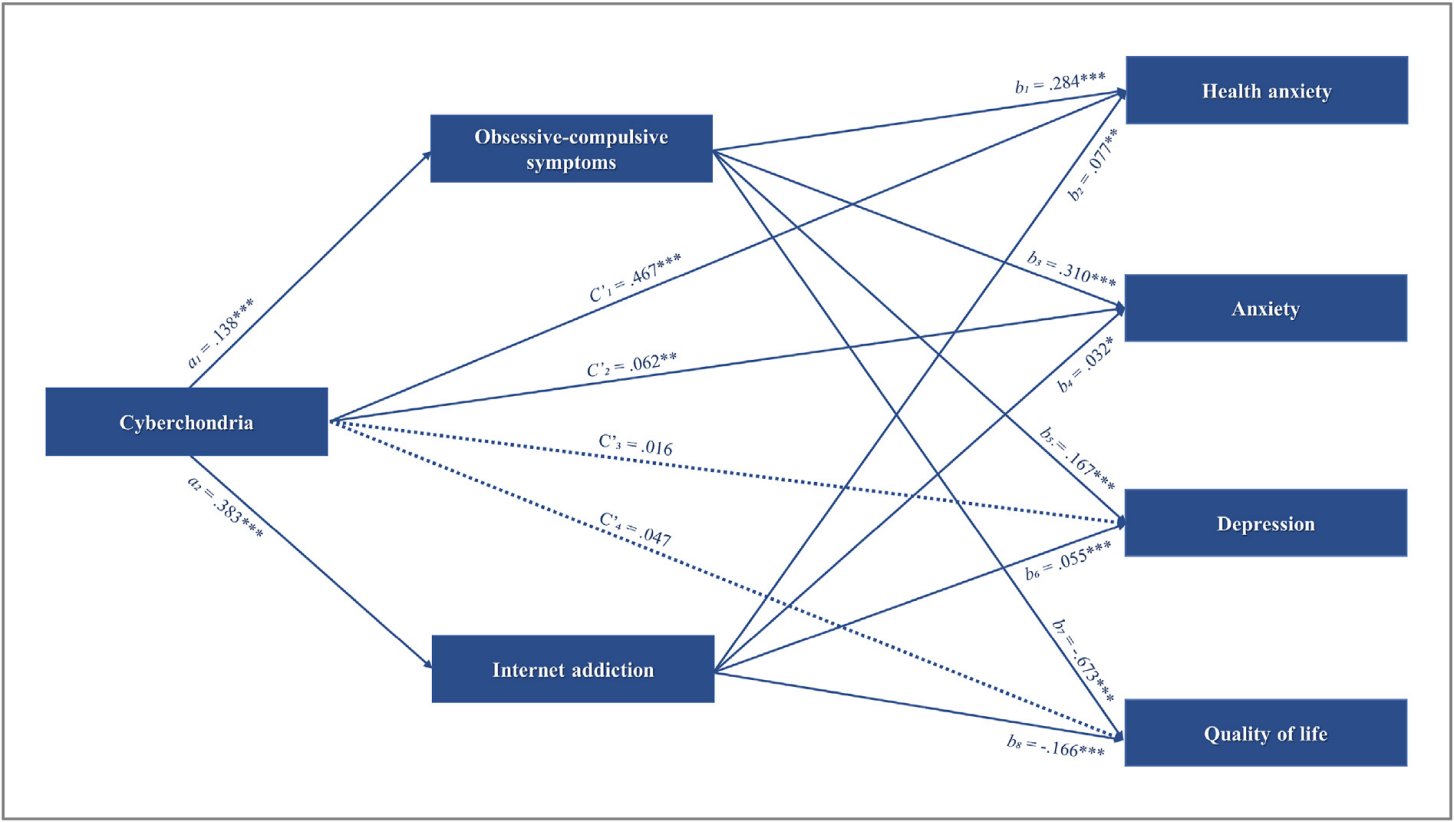
Statistical diagram of the parallel mediation model with Process Model 4. Path coefficient: unstandardized coefficient. Dashed line: nonsignificant path. ∗*p* < .05, ∗∗*p* < .01, ∗∗∗*p* < .001.

As can be seen in Figure 1, participants with higher levels of obsessive-compulsive symptoms (*b* = .138, SE = .020, *p* < 001) and Internet addiction (*b* = .383, SE = .059, *p* < 001) exhibited a more severe cyberchondria. Moreover, respondents with more severe obsessive-compulsive symptoms and Internet addiction showed higher levels of health anxiety (obsessive-compulsive *b* = .284, SE = .072, *p* < 001; Internet addiction *b* = .077, SE = .025, *p* = .002), anxiety (obsessive-compulsive *b* = .310, SE = .038, *p* < 001; Internet addiction *b* = .032, SE = .013, *p* = .013), depression (obsessive-compulsive *b* = .167, SE = .034, *p* < 001; Internet addiction *b* = .055, SE = .012, *p* < 001) and a worse quality of life (obsessive-compulsive *b* = -.673, SE = .118, *p* < 001; Internet addiction *b* = -.166, SE = .040, *p* < 001).

In addition, the bootstrap confidence intervals for the indirect effects based on 10,000 bootstrap samples were entirely above zero for health anxiety, anxiety and depression, whereas they were entirely below zero for quality of life (see Table 4). Specifically, obsessive-compulsive symptoms and Internet addiction partially mediated the link between cyberchondria and health anxiety and the link between cyberchondria and anxiety, whereas they fully mediated the link between cyberchondria and depression, and cyberchondria and quality of life. Thus, while cyberchondria was shown to influence health anxiety (*b* = .467, SE = .036, *p* < .001) and anxiety (*b* = .062, SE = .019, *p* = .001) independently from its effect on obsessive-compulsive symptoms and Internet addiction, there was no evidence that cyberchondria directly influenced depression (*b* = .016, SE = .017, *p* = .342) and quality of life (*b* = .047, SE = .058, *p* = .423).

**Table 4:** Bootstrapping indirect effect and 95% confidence interval for the model by Process Model 4.

	SHAI			HADS-A			HADS-D			WHOQoL		
	Coeff.	SE	CI	Coeff.	SE	CI	Coeff.	SE	CI	Coeff.	SE	CI
MOCQ-R	**.039**	.012	.017, .065	**.043**	.009	.027, .061	**.023**	.007	.011, .037	**-.093**	.024	-.144, -.051
IAT	**.030**	.013	.007, .057	**.012**	.006	.002, .024	**.021**	.006	.010, .035	**-.063**	.019	-.103, -.030

*Note.* Coeff, unstandardized coefficient of the indirect effect; SE, standard error; CI, 95% confidence interval based on 10,000 bootstrap samples. Significant mediations are shown in bold.

MOCQ-R, reduced form of the Maudsley Obsessional-Compulsive Questionnaire; IAT, Internet Addiction Test; SHAI, Short Health Anxiety Inventory; HADS-A, Hospital Anxiety and Depression Scale, Anxiety subscale; HADS-D, Hospital Anxiety and Depression Scale, Depression subscale; WHOQoL, WHO Quality of Life-BREF.

The overall models explained 42% of the total variance in health anxiety (F [7, 564] = 59.34, p < .001); 37% of the total variance in anxiety (F [7, 564] = 47.69, p < .001); 19% of the total variance in depression (F [7, 564] = 19.30, p < .001); 12% of the total variance in quality of life (F [7, 564] = 10.83, p < .001).

### 3.4 Moderating effect of COVID anxiety

**Figure 2:**
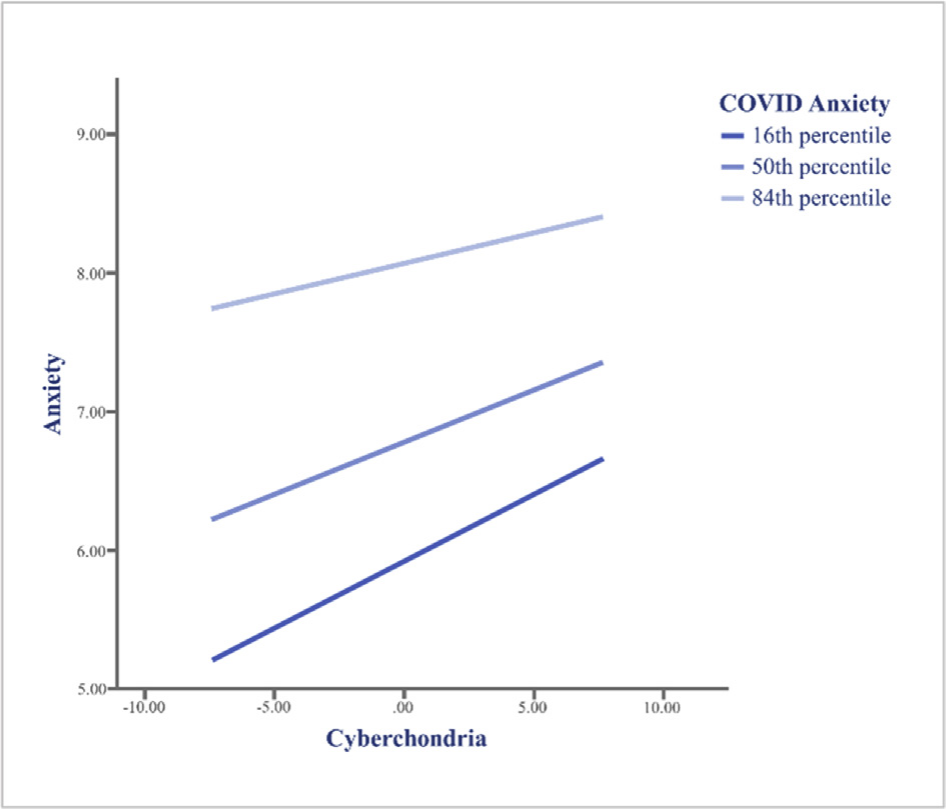
Conditional direct effect of cyberchondria on anxiety.

As can be seen in Figure 2, the result of the combined parallel mediation and moderation between cyberchondria and COVID anxiety obtained with Model 5 of Process showed that COVID anxiety moderates the relationship between cyberchondria and anxiety (*b* = -.010, SE .004, R^2^ change = .005, *p* = .020, 95% CI = [-.019, -.002]). The conditional effects of cyberchondria on anxiety were statistically significant at the 16th (b = .096, SE = .024, p < .001, 95% CI = [.050, .143]), 50th (b = .075, SE = .020, p < .001, 95% CI = [.037, .114]) and 84th (b = .044, SE = .020, p = .031, 95% CI = [.004, .084]) percentiles of COVID anxiety. The analysis of simple slopes revealed that the association between cyberchondria and anxiety was stronger for respondents with low COVID anxiety than for respondents with high COVID anxiety (see Figure 2).

No statistically significant interactions were found between cyberchondria and COVID anxiety with respect to health anxiety, depression and quality of life.

**Figure 3:**
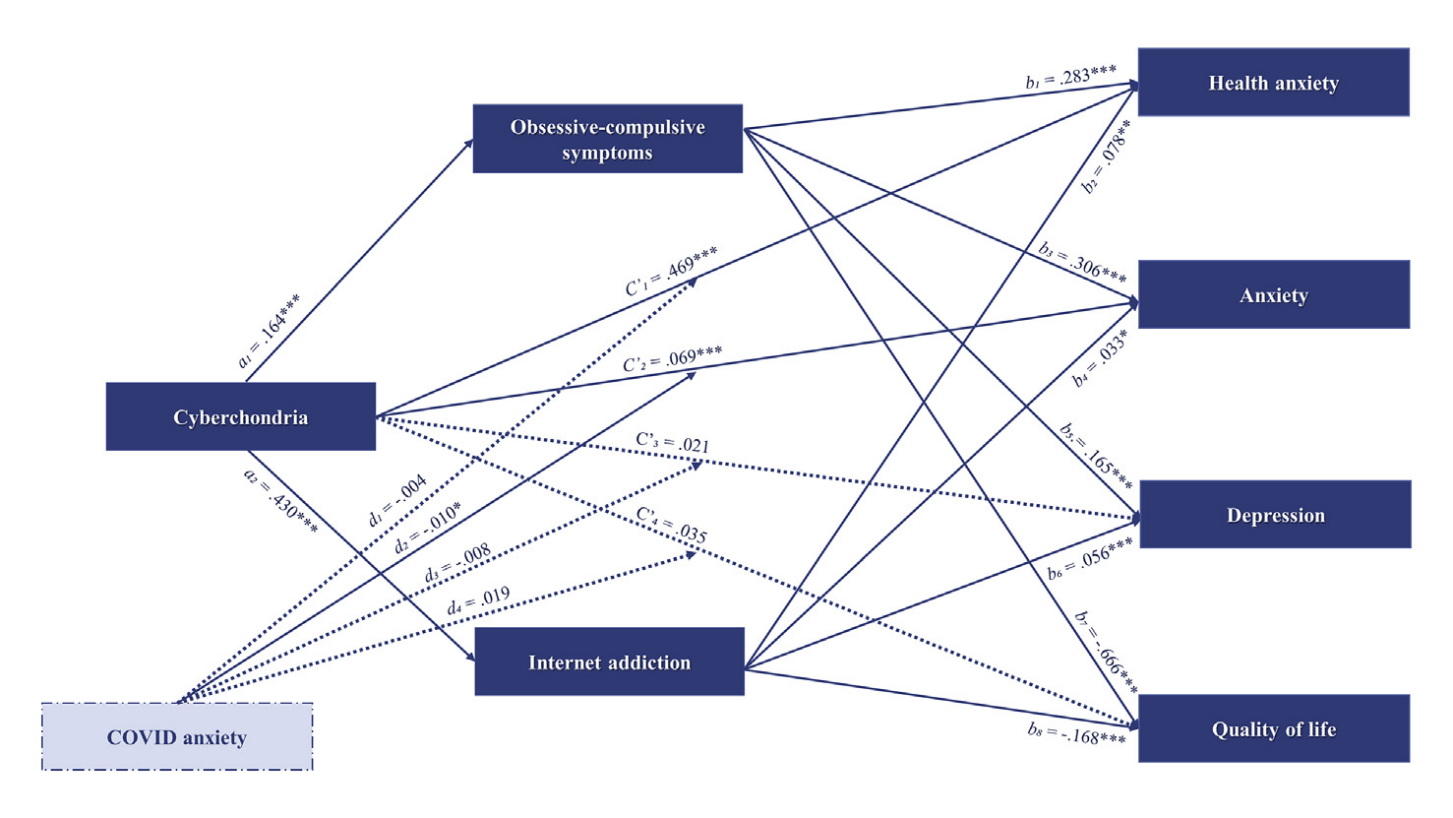
Statistical diagram of the model with combined parallel mediation and moderation between cyberchondria and COVID anxiety with Process Model 5. Path coefficient: unstandardized coefficient. Dashed line: nonsignificant path. ∗*p* < .05, ∗∗*p* < .01, ∗∗∗*p* < .001.

Furthermore, similar to Model 4, in Model 5 of Process we observed the partial parallel mediation effect of obsessive-compulsive symptoms and Internet addiction on health anxiety (indirect effects for health anxiety: obsessive-compulsive *b* = .046, bootstrap SE = .014, 95% bootstrap CI [.021, .076]; Internet addiction *b* = .033, bootstrap SE = .014, 95% bootstrap CI [.008, .063]) and anxiety (indirect effects for anxiety: obsessive-compulsive *b* = .050, bootstrap SE = .009, 95% bootstrap CI [.033, .070]; Internet addiction *b* = .014, bootstrap SE = .006, 95% bootstrap CI [.003, .027]), and the total mediation effect of obsessive-compulsive symptoms and Internet addiction on depression (indirect effects for depression: obsessive-compulsive *b* = .027, bootstrap SE = .007, 95% bootstrap CI [.014, .043]; Internet addiction *b* = .024, bootstrap SE = .006, 95% bootstrap CI [.013, .038]) and quality of life (indirect effects for quality of life: obsessive-compulsive *b* = -.109, bootstrap SE = .026, 95% bootstrap CI [-.165, -.062]; Internet addiction *b* = -.072, bootstrap SE = .021, 95% bootstrap CI [-.116, -.035]). The final model is shown in Figure 3.

## 4 Discussion

The present study aimed to understand the effect of cyberchondria on anxiety-depressive disorders and quality of life, via obsessive-compulsive symptoms and Internet addiction, during the COVID-19 pandemic. In our sample we found that obsessive-compulsive symptoms and Internet addiction partially mediated the relations between cyberchondria and health anxiety and between cyberchondria and anxiety, whereas they totally mediated the relationship between cyberchondria and depression, and between cyberchondria and quality of life.

Confirming the existing literature (Batıgün et al., 2020; Norr et al., 2015; Zangoulechi et al., 2018), we found that cyberchondria, as assessed by CSS-12, is significantly associated with more severe obsessive-compulsive symptoms and Internet addiction. Moreover, consistently with several studies conducted previously, even before the COVID-19 pandemic, higher scores in obsessive-compulsive symptoms and Internet addiction resulted associated with worse health anxiety (Fergus and Russell, 2016; Ivanova and Karabeliova, 2014), anxious-depressive symptoms (Batıgün et al., 2020; Hofmeijer-Sevink et al., 2018; Ko et al., 2012) and quality of life (Cheng and Li, 2014; Stengler-Wenzke et al., 2007; Tran et al., 2017).

Consistently with the previous literature on health anxiety (McMullan et al., 2019), a more severe cyberchondria proved directly associated with a greater health anxiety. Moreover, cyberchondria also directly predicted the generalized anxiety dimension, adding strength to the few research exploring the link between cyberchondria and anxiety other than health anxiety (Batıgün et al., 2020; Oniszczenko, 2021). In interpreting these findings, the partial overlap among the anxiety-spectrum constructs must be considered.

The significant indirect effects found in the present study highlight that both obsessive-compulsive symptoms and Internet addiction may have a central role in explaining the relationship between cyberchondria and different clinical outcomes.

Specifically, the indirect effects of cyberchondria on health anxiety and anxiety through obsessive-compulsive symptoms and Internet addiction seem to support studies which hypothesized that compulsive features may play a pivotal role in cyberchondria (Khazaal et al., 2021). However, it remains to be clarified whether the compulsive features characterizing the health-related online searches of cyberchondria must be considered as compulsive reassurance-seeking or as a more general compulsive Internet use, or both (Brown et al., 2020).

Regarding the absence of a direct link between cyberchondria and the depressive dimension, it can be hypothesized that the increasing *status* of alert, often experienced during health-related online information seeking (Doherty-Torstrick et al., 2016; White and Horvitz, 2009), tends to oppose and to prevail on the low mood characterizing depression. Considering previous literature on this topic, Starcevic et al. (2019) found that depressive symptoms were minimally related to cyberchondria, whereas Ivanova (2013) did not find a significant correlation between cyberchondria and depression. Interestingly, Arsenakis et al. (2021) found that depressive symptoms were negatively related to cyberchondria, with higher scores in depression predicting lower scores in cyberchondria. Our findings seem to suggest that cyberchondria affects depression in those individuals exhibiting compulsive problems. In support of this result, Gioia and Boursier (2020) highlighted that, although some cyberchondriac individuals might prove particularly engaged in reassurance-satisfying aspects of online health-related information research (for example attempting to answer health questions for themselves), the repeated anxiety-increasing behaviors may become addictive. The explanation is embedded in the key concept of compulsive Internet use, that is, that it self-damagingly persists notwithstanding the related distress (Khazaal et al., 2021). The present study suggests that cyberchondria, via an addictive component, increases depression. In general, addictive behaviors such as Internet addiction or Internet gaming behaviors are known to be strongly linked with depressive dimensions (Biolcati et al., 2021; Reed et al., 2017; Romano et al., 2013).

According to our results, cyberchondria does not directly predict a decreased quality of life. A possible explanation for this finding may lie in the fact that, during health-related online information searches, besides individuals experiencing anxiety, there are other individuals who find relief from online health information seeking (Starcevic, 2017; White and Horvitz, 2009), depending also on their pre-existing vulnerabilities (McMullan et al., 2019). However, this kind of relief may often persist only in the short term, needing further online searches for a long-term relief and triggering a compulsive mechanism of reassurance-seeking resulting in a loss of control (Taylor and Asmundson, 2004; Zheng and Tandoc, 2020). This could explain why, in our study, cyberchondria predicts a worsening of the quality of life only after taking into account the contribution of obsessive-compulsive symptoms and Internet addiction. It could be interesting for further research to examine whether, for different user search intents (i.e., query escalation, as studied by White and Horvitz, 2009) and considering the mediational role of compulsive features and health anxiety, cyberchondria predicts different outcomes in terms of quality of life.

Adding COVID anxiety as moderator, we found similar results to those obtained with the parallel mediation model. Thus, it does not change the predictive model of the relationship between variables, suggesting that cyberchondria may impact clinical variables beyond pandemic-related anxiety. COVID anxiety was found to moderate only the effect of cyberchondria on anxiety. Specifically, the cyberchondria-anxiety link was found to be stronger in respondents with low levels of COVID anxiety, whereas a high COVID anxiety seems to weaken the relationship between cyberchondria and anxiety. We hypothesize that, when an individual with high COVID anxiety searches for online health-related information, the seeking-behavior may be driven by the urgency to alleviate a specific type of anxiety (such as COVID anxiety) rather than a more generalized anxiety. However, since to our knowledge no other studies have examined these aspects, further research is needed.

Our findings need to be interpreted considering some limitations. First, the interpretation of our findings needs caution as our conclusion is less generalizable to the entire population. Indeed, given the restrictive measures implemented by the Italian government during the third wave of COVID-19, we used a snowball sampling technique for which there was an oversampling of some group of respondents (i.e., females, students) that makes less likely that the sample population reflects properly the actual pattern of the Italian general population. Moreover, the Internet-based recruitment may have cut out specific portions of the population (i.e., the elderly, people unfamiliar with technology), making the recruitment more accessible to individuals prone to the use of the Internet, who, anyway, may also be presumably more prone to search for health-related information on the web and, therefore, probably more suited for a study aimed to understand the underlying mechanisms of cyberchondria. Secondly, we collected data based on self-report measures which might be limited by self-report biases (i.e., social desirability). Moreover, data were collected during the COVID-19 third wave. Our models took into account COVID anxiety, but our study did not examine some aspects which proved to be associated with increased concern during pandemics, such as dysfunctional beliefs and interpretations, transdiagnostic processes, social, situational and environmental factors (Dennis et al., 2021). Lastly, given the cross-sectional nature of the present study and the fact that we are dealing with repetitive behaviors creating vicious cycles, it is impossible to establish causality directions among all the study's variables. Further research should focus on longitudinal studies to understand temporal precedence and determine whether cyberchondria leads to increased psychological symptoms or vice versa.

## 5 Conclusions

The present study highlights that obsessive-compulsive symptoms and Internet addiction contribute to explain the underlying mechanisms which link cyberchondria not only, as already noted, to increased health anxiety and anxiety, but also to increased depression and decreased quality of life. Our findings shed a light on an important aspect of cyberchondriac behavior, that is compulsivity. Furthermore, these results add information to the existing literature on cyberchondria, by exploring its associations with several psychological constructs often overlooked by previous studies, such as depression and quality of life.

From the point of interventions, clinicians should consider online compulsive health-related information seeking behaviors when dealing both with Internet addicted and with obsessive-compulsive patients, paying attention to aspects of cyberchondria in their diagnostic assessment. Moreover, healthcare practitioners should provide additional support for individuals with cyberchondria, taking into account the dimension of the compulsivity and its potential to worsen quality of life. Specific interventions should be provided to vulnerable groups, especially those with compulsive features, in order to empower them on how to approach and to handle online health information (Ünal et al., 2022). As health-related information, if properly used, may play a vital role in people's self-care, prevention efforts aimed to recognize not only the challenges, but also the opportunities of this type of information should be implemented to enhance people awareness about their own health and decisions about medical care (Kalantari et al., 2021).

From the point of the management strategies, previous studies have already pointed out as useful approaches the implementation of specific interventions by the governments to regulate web health information (Cuan-Baltazar et al., 2020), the improvement of search engine features (i.e. Internet searches ranking criteria) combined with a 'health information literacy' of online health-information seekers and a better comprehension of the reasons underlying the preference for certain health websites and the avoidance of others (Starcevic and Berle, 2013). Supporting the validity of these indications, based on our findings, we suggest as a further useful step, the understanding of which kinds of health-related contents tend to elicit a higher response in terms of web-search compulsivity, considering the sources (media vs. professional practitioners), the topics (e.g., cancers, rare diseases, illnesses highly prompted by media) and the way they are disclosed on websites (e.g., simply mentioning the correlation between certain symptoms and severe illnesses, or highlighting the probability rates). Finally, considering the current pandemic context, governments may have a fundamental role. While the evidence seem still controversial concerning the impact of the timeliness and the stringency of governments’ response to COVID-19 on physical and mental health (Ćepulić et al., 2021; Chen et al., 2021a, 2021b; Lee et al., 2021a, 2021b; Long et al., 2021), higher levels of trust in governments seem to increase the adoption of protective behaviors among the public (Robinson et al., 2021) and to moderate the risk perception of COVID-19 (Xu, 2021) and the psychological distress (Ahn et al., 2021; Olagoke et al., 2020), even when restrictions are stringent (Tan et al., 2021). As misinformation showed to be negatively associated with lower levels of confidence in governments and scientific institutions (Pickles et al., 2021), it is fundamental that governments spread consistent and clear messages and knowledge on COVID-19 to help people to deal with this kind of information and to identify trustworthy sources (Okan et al., 2020), moderating the detrimental impact of the infodemic on psychological wellbeing (Samal, 2021).

## Declarations

### Author contribution statement

Federica Ambrosini; Roberto Truzoli; Matteo Vismara; Daniele Vitella; Roberta Biolcati: Conceived and designed the experiments; Performed the experiments; Analyzed and interpreted the data; Contributed reagents, materials, analysis tools or data; Wrote the paper.

### Funding statement

This research did not receive any specific grant from funding agencies in the public, commercial, or not-for-profit sectors.

### Data availability statement

Data will be made available on request.

### Declaration of interests statement

The authors declare no conflict of interest.

### Additional information

No additional information is available for this paper.

## Appendix A Supplementary data

Supplementary contents.docx Supplementary contents.docx
