# Supplementary material for: The effect of cyberchondria on anxiety, depression and quality of life during COVID-19: the mediational role of obsessive-compulsive symptoms and Internet addiction
Source: Heliyon. 2022 May 14;8(5):e09437. doi: 10.1016/j.heliyon.2022.e09437 (PMC9107336; doi:10.1016/j.heliyon.2022.e09437)
Supplement: Supplementary contents.docx [file mmc1.docx]

# Supplementary contents

## Preliminary regression analysis

In hierarchical regression n. 1, 2, 3 and 4, COVID anxiety, gender, age, the occupational status, physical and psychiatric comorbidities were entered as controlled variables in the first step of the regression; in step 2 cyberchondria was entered as predictor and in step 3 obsessive-compulsive symptoms and internet addiction were entered as mediators. In hierarchical regression n. 5, 6, 7 and 8, gender, age, the occupational status, physical and psychiatric comorbidities were entered as controlled variables in the first step of the regression; in step 2 cyberchondria, COVID anxiety and the interaction term cyberchondria x COVID anxiety were entered as predictors and in step 3 obsessive-compulsive symptoms and internet addiction were entered as mediators. In hierarchical regressions n. 1 and 5 the dependent variable was health anxiety; in n. 2 and 6 was anxiety; in n. 3 and 7 was depression; in n. 4 and 8 was the quality of life.

Results of the hierarchical regression 1, 2, 3 and 4 showed that all the models significantly explained the variance of the dependent variables (SHAI: *R^2^* = .45, *F* _9, 562_ = 52.064, *p* <.001; HADS-A: *R^2^* = .45, *F* _9, 562_ = 51.791, *p* <.001; HADS-D: *R^2^* = .27, *F* _9, 562_ = 22.936, *p* <.001; WHOQoL: *R^2^* = .21, *F* _9, 562_ = 16.138, *p* <.001). Equally, also results of the hierarchical regression 5, 6, 7 and 8 showed that all the models significantly explained the variance of the dependent variables (SHAI: *R^2^* = .46, *F* _10, 562_ = 46.821, *p* <.001; HADS-A: *R^2^* = .46, *F* _10, 562_ = 47.530, *p* <.001; HADS-D: *R^2^* = .27, *F* _10, 562_ = 21.094, *p* <.001: WHOQoL: *R^2^* = .21, *F* _10, 562_ = 14.740, *p* <.001).

All the Durbin-Watson values converged to 2.0 indicating independence of residuals (Rutledge and Barros, 2001). The Variance Inflation Factors (VIF) are all < 5 indicating the absence of strong multicollinearity (Akinwande et al. 2015). The visual inspection of scatterplots (see Figure S1) does not suggest the presence of problematic heteroscedasticity. Considering that relatively mild heteroscedasticity does not cause profound problems in regression (Hayes and Cai, 2007), the homoscedasticity assumption was considered satisfied. The visual inspection of histograms (Figure S2) and P-P plots (Figure S3) suggests a fairly normal distribution of residuals. Considering that only the most severe violations of the normality assumption substantially affect the validity of statistical inferences from a regression analysis (Hayes, 2018), also the normality assumption was considered met.


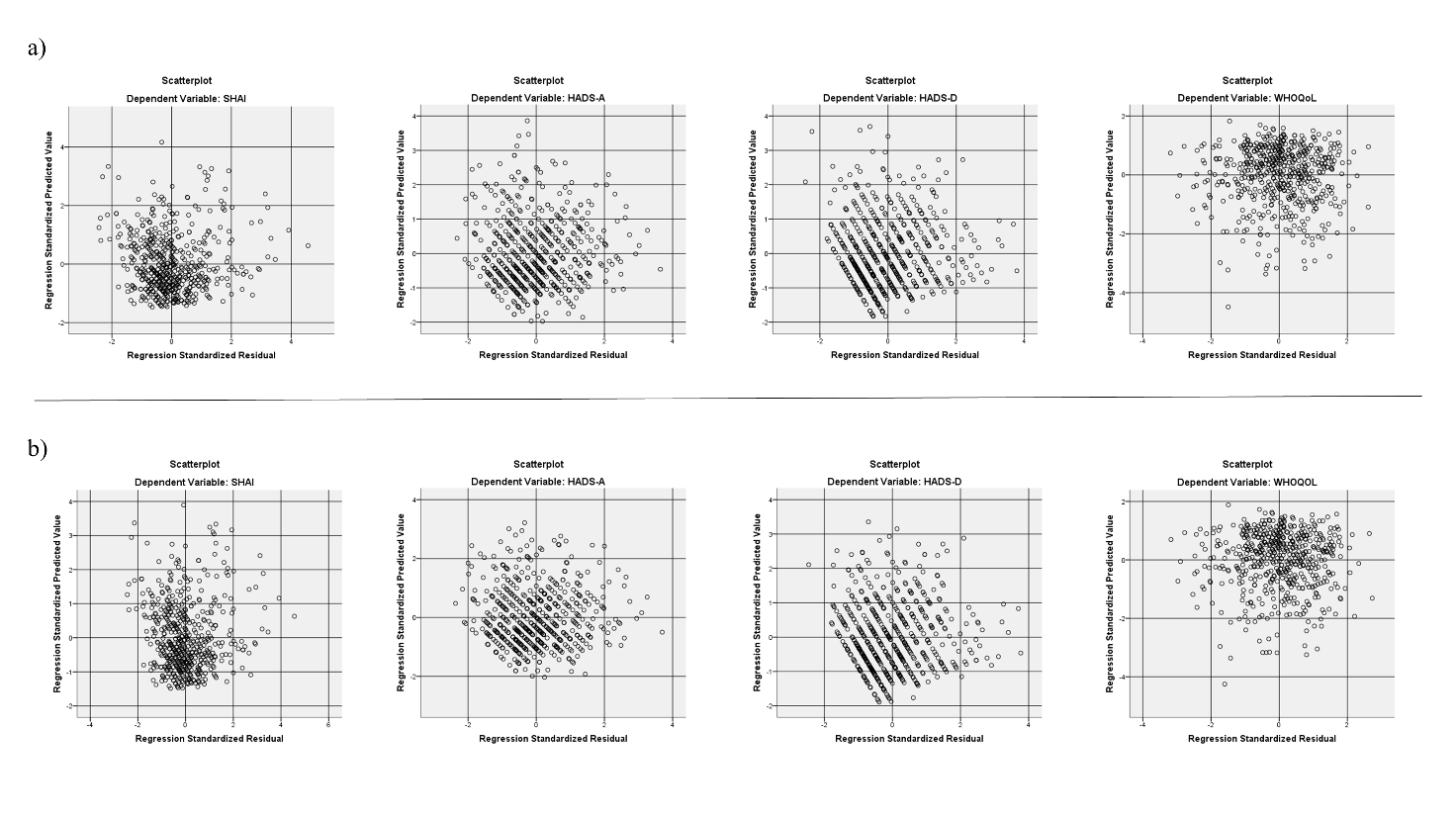


*Figure S1. Scatterplots of Standardized Residuals vs Standardized Predicted Values from hierarchical regressions n. 1, 2, 3, 4 (a) and hierarchical regressions n. 5, 6, 7, 8 (b).*


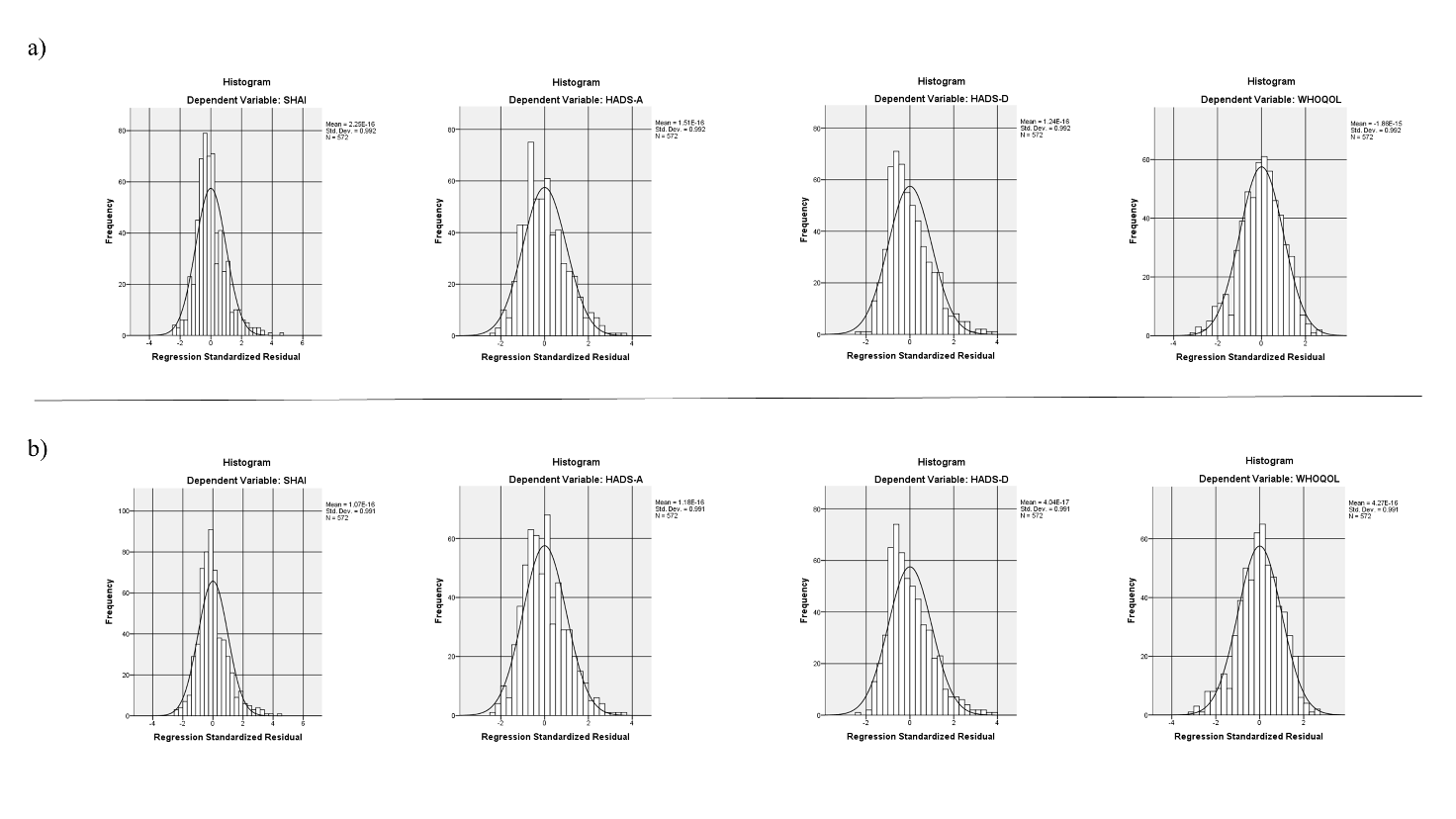


*Figure S2. Histograms of the residuals from hierarchical regressions n. 1, 2, 3, 4 (a) and hierarchical regressions n. 5, 6, 7, 8 (b).*


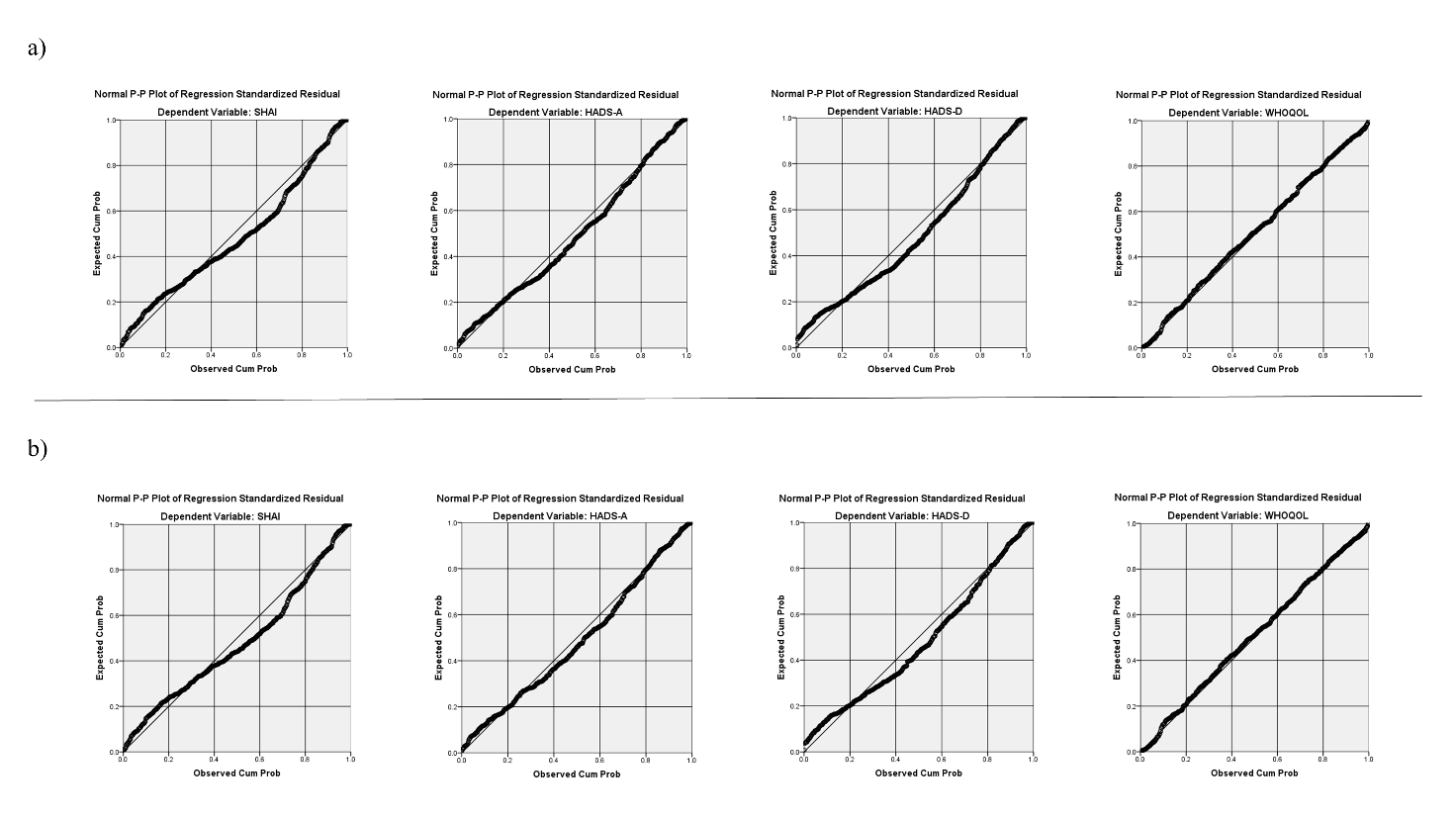


*Figure S3. P-p plots of standardized residuals from hierarchical regressions n. 1, 2, 3, 4 (a) and hierarchical regressions n. 5, 6, 7, 8 (b).*

**References**

Akinwande, M. O., Dikko, H. G., & Samson, A. (2015). Variance inflation factor: as a condition for the inclusion of suppressor variable (s) in regression analysis. *Open Journal of Statistics*, *5*, 754. https://doi.org/10.4236/ojs.2015.57075

Hayes, A. F., & Cai, L. (2007). Using heteroskedasticity-consistent standard error estimators in OLS regression: An introduction and software implementation. *Behavior research methods*, *39*(4), 709-722.

Hayes, A.F. (2018). Introduction to Mediation, Moderation, and Conditional Process Analysis: A Regression-based Approach, 2nd Edition. ed. Guilford Press.

Rutledge, D. N., & Barros, A. S. (2002). Durbin–Watson statistic as a morphological estimator of information content. *Analytica Chimica Acta*, *454*(2), 277-295. https://doi.org/10.1016/S0003-2670(01)01555-0
